# Auditory rhythm complexity affects cardiac dynamics in perception and synchronization

**DOI:** 10.1371/journal.pone.0293882

**Published:** 2023-11-17

**Authors:** Shannon E. Wright, Caroline Palmer

**Affiliations:** Department of Psychology, McGill University, Montréal, Québec, Canada; University of Pecs Medical School, HUNGARY

## Abstract

Accurate perception and production of auditory rhythms are key for human behaviors such as speech and music. Auditory rhythms in music range in their complexity: complex rhythms (based on non-integer ratios between successive tone durations) are more difficult to perceive and produce than simple rhythms (based on integer ratios). The physiological activity supporting this behavioral difference is not well understood. In a within-subjects design, we addressed how rhythm complexity affects cardiac dynamics during auditory perception and production. Musically trained adults listened to and synchronized with simple and complex auditory rhythms while their cardiac activity was recorded. Participants identified missing tones in the rhythms during the Perception condition and tapped on a keyboard to synchronize with the rhythms in the Synchronization condition. Participants were equally accurate at identifying missing tones in simple and complex rhythms during the Perception condition. Tapping synchronization was less accurate and less precise with complex rhythms than with simple rhythms. Linear cardiac analyses showed a slower mean heart rate and greater heart rate variability during perception than synchronization for both simple and complex rhythms; only nonlinear recurrence quantification analyses reflected cardiac differences between simple and complex auditory rhythms. Nonlinear cardiac dynamics were also more deterministic (predictable) during rhythm perception than synchronization. Individual differences during tapping showed that greater heart rate variability was correlated with poorer synchronization. Overall, these findings suggest that linear measures of musicians’ cardiac activity reflect global task variability while nonlinear measures additionally reflect stimulus rhythm complexity.

## Introduction

Listeners are adept at hearing and reproducing a wide range of auditory rhythms, such as those contained in dance steps or in musical melodies. Auditory rhythms are composed of sequences of regularly spaced events over time [1]. Some rhythms–those defined by complex ratios between the sequential tone durations—are less common and more difficult to perceive and produce [2–5]. Behavioral differences between simple and complex rhythms and the physiological activities that support them are not well-understood. Auditory rhythms such as musical rhythms are known to influence cardiac activity; previous research has largely focused on how acoustic features of musical rhythms, such as tempo or pitch, affect cardiac activity during perception [6]. A few studies have shown changes in the periodicities present in cardiac rhythms during perception [7] and production [8] of different auditory rhythms. Yet little research has addressed the specific relationship between auditory rhythm complexity and physiological activity. The current study investigates how auditory rhythm complexity affects behavior and cardiac dynamics during perception and production, using a synchronization paradigm.

Changes in heart rate mean and variability during cognitive-motor tasks are often attributed to changes in physiological arousal and task difficulty [9–11]. Slower heart rate and greater heart rate variability tend to correspond to lower arousal states [12]. Both musical acoustic features and musical task difficulty can influence cardiac rhythms. In perception-only tasks, some studies show that fast-tempo musical rhythms tend to increase listeners’ mean heart rate and decrease their heart rate variability, indicative of an arousal effect [6, 13–16, cf. 17]. Other studies show that faster-tempo auditory rhythms tend to elicit faster heart rates in listeners without changes in heart rate variability [16, 18–20]. Slow-tempo music has been shown to slow down listeners’ heart rates and increase heart rate variability [21, 22]. Task factors in music performance also influence physiological arousal: Performing music in front of an audience compared to performing alone increased a professional musician’s heart rate and decreased heart rate variability [23]. Musical task familiarity can influence cardiac rhythms: Pianists’ (≥ 6 years private piano instruction) heart rates increased during production of unfamiliar (more challenging) musical melodies compared with familiar melodies and a silent baseline condition [8]. Together, these studies suggest that auditory rhythms such as those found in music modulate listeners’ arousal levels, with task difficulty potentially contributing to this effect during rhythm production. Thus, we expected that changes in task difficulty based on auditory rhythm complexity might impact heart rate and heart rate variability during perception and synchronization, such that simple rhythms result in slower heart rate and greater heart rate variability than complex rhythms.

Nonlinear dynamical systems theory provides an explanation for the effects of rhythm complexity in music perception and production. Oscillators (signals with recurring cyclic patterns with a natural or default frequency) are central in this theory: Rhythm complexity depends on the amount of coupling or interaction between two oscillators which is determined in part by the ratio of one oscillator frequency to the other oscillator frequency. Oscillators whose natural (default) frequencies form small integer ratio relationships, such as 1:2, show stronger coupling, compared to oscillators whose natural frequencies form non-integer ratios, such as 3:2 [24–26]. In this study, we compare Simple rhythms whose tone durations form small integer ratios (1:2) with Complex rhythms whose tone durations form non-integer ratios (3:2). Neural resonance theory extends this nonlinear dynamical systems perspective specifically to auditory rhythm perception, holding that rhythm perception is underpinned by internal neural oscillations that couple with external auditory rhythms; when neural oscillations and auditory rhythms form simple integer ratios, coupling is most likely to occur [24, 25]. The duration ratio distinction of simple and complex rhythms converges with auditory rhythm structures that are most frequently observed across musical cultures [27], and recent evidence suggests that small integer ratios primarily characterize the structure of certain non-human animal vocalizations [28, 29].

Production of auditory rhythms by humans supports the dynamical systems distinction between Simple and Complex rhythms. Individuals reproduced auditory rhythm sequences better when the successive tone intervals formed integer ratios such as 2:4 compared with 2:3 [2]. When asked to tap one hand in synchrony with non-integer ratio auditory rhythms, individuals with a range of musical experience tended to distort their taps toward simple integer ratios [4, 5, 30]. A similar effect has been found when individuals tap both hands with Simple and Complex polyrhythms (rhythms that are composed of more than one tone sequence) [31]. In an iterated learning paradigm in which individuals initially heard and reproduced random sequences of tones, individuals produced sequences that converged toward small integer ratio relationships between tones [32]. There is also some cross-cultural evidence for a bias toward producing simple auditory rhythms [33], although cultural familiarity with different musical rhythms may modulate these biases in both rhythm production [34] and rhythm perception [35].

A few studies directly assess Simple and Complex auditory rhythms in perception and production tasks. Repp et al. [36] measured musicians (graduate students in music performance) as they listened to and tapped in synchrony with simple and complex rhythms that contained temporal perturbations. They found evidence of better perception and production for small-integer ratio rhythms (such as 1:1, 1:2 ratios) than for complex rhythms (such as 4:5, 7:11, 5:13), suggesting similar constraints on rhythm perception and production. However, there was no clear advantage across perception and production for all of the small integer ratios used in the experiment [36]. As most studies address perception or production tasks but not both, it is unknown whether the tendency toward Simple over Complex rhythms is stronger in perception or production.

Neurophysiological studies have investigated how rhythm complexity affects oscillatory neural activity. Mathias et al. [37] directly compared neural oscillations with EEG while musicians ((≥ 6 years private musical instruction) perceived and produced auditory stimuli containing simple (1:1), moderate (1:2), and complex (3:2) rhythms. During perceptual trials, participants detected omitted tones in the auditory rhythms; during production trials, participants tapped the second part of the ratio of each auditory rhythm to produce the full rhythm. Detection of missing tones was most accurate for the 1:1 rhythm with no difference observed between the 1:2 and 3:2 rhythms. Tapping was more accurate and precise for the 1:1 and 1:2 rhythms compared to the 3:2 rhythms. Power spectral density measures (EEG) were greatest at frequencies that corresponded to the tapping frequency in the simple (1:1) rhythm compared to the moderate (1:2) and complex (3:2) rhythms [37]. These findings suggest that neurophysiological entrainment is stronger for simple rhythms than complex rhythms, particularly during production (synchronization) tasks compared to perceptual tasks.

One of the goals of the current study is to compare cardiac rhythms during auditory rhythm perception and production tasks in a synchronization paradigm. Previous studies of cardiac activity during rhythm perception and production tend to employ linear measures of cardiac activity, such as mean heart rate or R-R interval and heart rate variability. For example, one study reported no difference in linear measures of listeners’ heart rate variability during music perception of duple metre rhythms (a march) compared to triple metre rhythms (a waltz) [7]. Linear measures such as heart rate variability assume that the cardiac signal is stationary, meaning the mean and variance of the time series are relatively stable over time [38]. In reality, stationarity in cardiac activity is often not the case [39]; behavioral tasks are likely to have different effects on cardiac activity over time. Thus, it is possible that nonlinear cardiac measures, which address relationships within a single time series, may be more sensitive to rhythmic changes that take place within musical stimuli. Indeed, previous research has successfully applied the nonlinear analysis technique of multiscale sample entropy, a measure of the predictability of a signal in time, to performing musicians’ cardiac dynamics. Professional musicians showed more predictable, less complex cardiac dynamics during higher-stress music performances [23, 40] and during jazz music performance compared to classical music performance [41].

Recurrence quantification analysis (RQA) is a nonlinear analysis alternative that does not make assumptions about stationarity of the signal [42]. In RQA, a time-delayed copy of the cardiac time series is produced to reconstruct the signal in a multi-dimensional phase space [43, 44]. Points in the phase space are then assessed for their closeness; points that are sufficiently close to one another in the phase space are deemed to be recurrent [45]. These recurrent points are plotted in a 2-dimensional recurrence plot and quantified according to different metrics to describe the behavior of the system. RQA has been applied to identify disrupted cardiac dynamics in clinical populations such as syncope (fainting) [46, 47], sleep apnea [48], and ventricular tachyarrhythmia [49] as well as changes in cardiac rhythms that occur during behavioral tasks. Konvalinka et al. [50] reported increased predictability and stability in cardiac dynamics of firewalkers during a ritual fire walk compared to spectators’ cardiac dynamics. Wright and Palmer [8] reported increased predictability in pianists’ (≥ 6 years private piano instruction) cardiac dynamics when they performed simple melodies compared to a silent baseline; furthermore, pianists’ cardiac dynamics were most predictable when they performed novel (unfamiliar) melodies. It is unknown, however, how much cardiac rhythms change during perception or production of auditory rhythms and whether auditory rhythm complexity affects cardiac dynamics.

The current study investigated how auditory rhythm complexity affects cardiac dynamics during auditory perception and synchronization tasks. The first aim was to determine whether rhythmic complexity, measured by the ratio of tone durations in two simultaneously presented rhythms, influences behavior (perception and synchronization) and of cardiac dynamics. The second aim was to compare how listening to auditory rhythms versus actively synchronizing a voice or part with those rhythms affects behavior and cardiac dynamics. Trained musicians (adults with ≥ 6 years private instruction on a musical instrument), who have had experience synchronizing their movements to rhythmic sound, listened to and tapped in synchrony with simple and complex auditory rhythms. Based on previous studies, our initial behavioral hypothesis was that tapping performance should be worse for the Complex rhythm (3:2 duration ratios) than for the Simple rhythm (1:2 duration ratios) [4, 5, 30, 37]. Also based on previous findings [8], our initial cardiac hypotheses were that greater recurrence and predictability of cardiac dynamics should be obtained during the synchronization task than during the perception task, and greater predictability should be obtained during the Complex rhythm compared to the Simple rhythm (across perception and synchronization tasks). A final aim was to test the hypothesis that individuals’ behavioral measures of auditory rhythm complexity would correspond to their cardiac measures, which we tested with simple correlations.

## Methods

### Participants

Twenty-five musically trained adults participated in the study (mean age = 22.24 years, sd = 3.78, N_female_ = 20). Participants had an average of 13.5 years of private musical instruction (range = 6–18 years; sd = 3.88 years). Participants were screened by email for eligibility; eligible participants had to be between 18 and 35 years of age and have at least 6 years of private musical instruction. Exclusion criteria included a history of cardiovascular, respiratory, neurological, or psychiatric disorders, a diagnosed hearing impairment, or having taken a transcontinental flight within 3 weeks of participating in the study. Participants were screened for normal hearing for the range of tone frequencies used in the auditory stimuli (< 30 dB HL threshold for 125–750 Hz frequencies), as determined by an audiometry screening at the beginning of the experiment. Participants were recruited via social media and flyer postings between May and October 2022; 10 participants were recruited for course credit in the Psychology Department at McGill University and 15 participants were recruited from the general adult population in Montreal. Individual participant identifiers available at the time of testing were removed following their participation. The study took place at McGill University and the protocol was reviewed by the Research Ethics Board (Ethics protocol #197–1018).

### Stimulus materials and equipment

**Fig 1:**
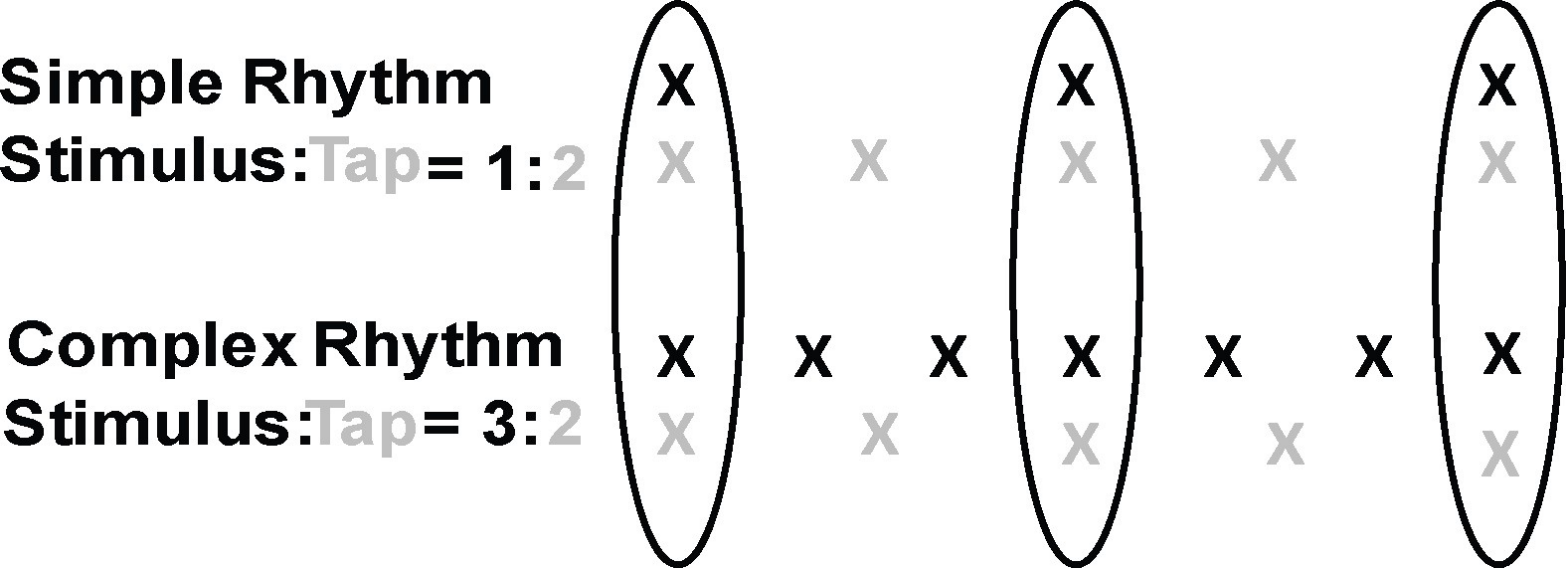
Simple and complex auditory rhythms used in the perception and synchronization tasks. Black and grey tones were sounded in the Perception condition. Black indicates sounded tones and grey indicates participants’ instructed taps that produced tones in the Synchronization task. Circles indicate stimulus beats on which asynchronies between sounded tones and produced taps were computed.

The auditory rhythms in all conditions were composed of two isochronous sequences of tones that differed in pitch and duration and formed specific temporal ratios with one another (Fig 1), similar to Mathias et al. [37]. The high-pitched tones were presented as 660 Hz sine tones and the low-pitched tones were presented as 392 Hz woodblock tones produced by a Roland Sound Canvas (SC-55) tone generator (timbre = 116). The high-pitched and low-pitched tones in the Simple rhythm formed a 1:2 ratio such that the high-pitched tone (intertone interval = 1090 ms) occurred once for every two low-pitched tones (intertone interval = 545 ms). The high-pitched and low-pitched tones in the Complex rhythm formed a 3:2 ratio such that the high-pitched tone (intertone interval = 363.33 ms) occurred three times for every two low-pitched tones (intertone interval = 545 ms). Thus, the low-pitched tone interonset interval (545 ms) was constant across both the Simple and the Complex rhythms; only the high-pitched tone interval changed across the rhythms. Each auditory rhythm was sounded for 1 minute and began with four woodblock tones (intertone interval = 545 ms). Each 1-minute auditory rhythm was repeated five times with a silent pause of 10 seconds between each repetition. Pilot testing with longer trials (five minute duration) generated a sufficient number of tapping errors as individuals stopped and restarted, which had the outcome of creating missing data in a time series analysis. Five one-minute trials with short (10s) gaps inbetween were thus chosen to balance the length of the physiological time series with the accuracy of behavioural performance.

Auditory stimuli in the Perception condition contained one omitted tone in either the high-pitched or low-pitched tone stream on two of the five trials for each rhythm (Simple and Complex) that participants were asked to detect. Tone omissions were placed at either the middle or the end of the rhythm sequence; trials with tone omissions were randomized across rhythm conditions and participants. Auditory stimuli in the Synchronization condition contained only the high-pitched tone; each auditory rhythm began with four woodblock tones (presented with the same timbre as in the Perception condition) that served as the participants’ tempo cue for tapping the low-pitched tone part (set to 545 ms). Thus, the participants’ tapping rate in the Synchronization task was constant across all trials of the Simple and Complex rhythms.

Sound was delivered through AKG K271 Studio headphones and participants tapped their finger on a Roland RD-700 electronic piano keyboard. Auditory stimuli and participants’ auditory feedback were generated on the Roland Sound Canvas (SC-55) tone generator using MIDI (musical instrument digital interface) with 1-ms temporal resolution. Participant key taps were recorded in FTAP v.2.1.07b [51] on a Dell T3600 PC running Linux (Fedora 16). Cardiac activity was recorded with a Polar H10 chest strap heart rate monitor with 1-ms temporal resolution connected via Bluetooth to the application EliteHRV running on an iPad Mini. Questionnaires included a musical background questionnaire and a short questionnaire about participants’ physical activity in the hour prior to the experimental session.

### Design

Behavioral performance and cardiac activity were measured for all participants in all auditory rhythm conditions, making this a within-subjects Task (Perception and Synchronization) by Rhythm (Simple vs Complex) design. In addition, each participant had a 5-minute silent baseline measure of cardiac activity collected. The order of conditions in the experiment was kept constant across participants, such that all participants first completed the physiological baseline measurement followed by auditory rhythm perception, then synchronization. The Synchronization condition followed the Perception condition to avoid unintentional motor imagery or motor planning during the perceptual task. The Simple rhythm condition always preceded the Complex rhythm condition so that participants began with the easier task.

Dependent variables for the Perception task were hit rate (% correct detection) and false alarm rate (% incorrect detection) for the omitted tones. The dependent variables for the Synchronization task were the mean intertap interval (ITI) as well as the tapping accuracy (participant tap onset time–stimulus tone onset time) for participant taps that coincided with stimulus tones, indicated with circles in Fig 1. Tapping precision was measured by the standard deviation of the mean signed asynchronies. Dependent variables for the cardiac signals included mean R-R intervals (normal heart beat-to-heart beat intervals, ms) and the root mean square of successive R-R interval differences (RMSSD, ms), a measure of heart rate variability [52]. Nonlinear cardiac measures included Recurrence Rate (% Rec) and Determinism (% Det), described below (Data Analysis).

### Procedure

Participants were invited to the lab to complete the 1-hour in-person testing session between 09h and 17h. Upon arrival at the lab, participants provided written informed consent by reading and signing a consent form and then completed an audiometric screening in which pure tones were presented over sound-attenuating headphones (Maico MA40). Only participants who reported hearing the range of tones used in the experiment (125 Hz– 750 Hz) at an average threshold of < 30 dB continued in the experiment.

Next, a 5-minute baseline cardiac recording was conducted. Participants attached the heart rate monitor around their chest and sat in a comfortable chair with their legs uncrossed. During the baseline recording, participants completed written questionnaires with minimal body movement. Participants then completed the Perception task. They were told that each rhythm they heard would have a high-pitched part and a low-pitched part and were asked to identify whether a trial had a missing tone by circling “yes” or “no” on a sheet of paper at the end of each trial. Participants listened to an example of a rhythm with and without a missing tone in initial practice trials for each rhythm. Participants completed five 1-minute Perception trials of the Simple (1:2) rhythm followed by five 1-minute perception trials of the Complex (3:2) rhythm.

Participants then completed the Synchronization task. They were asked to tap the low-pitched part of the rhythm on a single key of the piano keyboard using their dominant hand, so that their taps synchronized with the high-pitched part of the rhythm to form the intended rhythm ratio in each condition. Participants heard the high-pitched part of the rhythm as well as their own taps (low-pitch part of the rhythm) during the Synchronization condition. Participants received up to three practice trials tapping the rhythm before completing five 1-minute Synchronization trials for each rhythm condition. At the end of the Synchronization task, participants removed the heart rate monitor and were debriefed. The experimental session took approximately 1 hour to complete.

### Data analysis

#### Behavioral data

Each participant’s tapping data formed a time series of intertap intervals. Tap ITIs were examined for double taps (< 75 ms between two successive taps) and, when present (approximately 1.4% of all taps), the second tap was removed. The first 4 taps (indicating initial synchronization) and the last 4 taps were then removed from each trial, similar to Mathias et al. [37]. Intertap intervals greater than 3 standard deviations from the mean ITI were removed (Simple rhythm = 0.67% of all intervals; Complex rhythm = 0.65% of all intervals) and the mean ITI and CV were then calculated for each trial using Matlab (version 9.8.0, 2020). The mean absolute asynchrony, mean signed asynchrony, and standard deviation of mean signed asynchrony were calculated in each trial for each tap that aligned with a stimulus event, as shown by the circles in Fig 1. Asynchrony values were generated by subtracting the stimulus tone onset time from the participant tap onset time [53]. Smaller absolute asynchrony values indicate more accurate tapping; negative mean signed asynchrony values indicate participants’ taps anticipated the stimulus tone. A negative mean asynchrony between stimulus and response is commonly observed in auditory-motor synchronization tasks [54] such as the one used in the current study, and the measure captures phase synchronization between a tap and an auditory stimulus. A smaller standard deviation of the mean signed asynchronies indicates more precise tapping.

#### Cardiac data

Cardiac data were processed in Kubios HRV Premium (version 3.5.0). R-R interval series were generated for each trial, and mean R-R interval and RMSSD were calculated in Kubios. The RMSSD is primarily a measure of vagally-mediated beat-to-beat heart rate variability [55]. It was deemed an appropriate measure of heart rate variability as there is some evidence that RMSSD measures at rest are robust to differences in respiration rate [56, 57]. RMSSD has also been validated on 60-second durations taken from and compared with five-minute cardiac recordings [58], the same trial durations used in the current study.

The R-R values were then converted into beats per minute (BPM) that resulted in values every 300 ms (ie. measures faster than the maximal heart rate of all participants), following Wallot et al. [59]. Each R-R value in the time series (indicating onset times) was replaced with a string of BPM values. The BPM values were then averaged using a non-overlapping moving window of 300 ms, and the resultant time series served as input to the recurrence quantification analysis (RQA). The result of this transformation is an upsampling of the time series that creates a time series of 180 samples per trial, consistent across trials and participants.

Auto recurrence quantification analysis (RQA) was applied to the cardiac time series and was performed in Matlab with the CRP Toolbox 5.22 [60]. In contrast to the linear cardiac measures, RQA captures recurring patterns in cardiac activity over time. RQA metrics have been shown to yield robust statistics for physiological time series as short as 100 data points [61], which are shorter than those used in the current study (180 samples). Symmetrical, binary (recurrent, non-recurrent) recurrence plots were generated, which capture the recurring patterns in the cardiac signal (see Fig S1 in S1 File). Recurrence plots were generated on normalized values of the cardiac timeseries. Two metrics were used to quantify the recurrence patterns observed in the recurrence plots. The first was Recurrence Rate, a measure of the total proportion of recurrent to non-recurrent points. The second was Determinism: The proportion of sequential recurrent points, corresponding to predictability of the cardiac signal. For a detailed description of RQA methodology and chosen parameters, see S1 Appendix in S1 File.

Statistical tests for the behavioral and physiological measures were conducted in R Studio (version 4.2.0). Repeated-measures analyses of variance (using the function *ezANOVA* from the *ez* package in R) were used to investigate differences between more than two conditions, with a p-value below .05 determining statistical significance. Planned contrasts with the Holm-Bonferroni correction were used to follow-up significant analyses of variance. Trial-level participant data was the input for all statistical tests. The number of participants was chosen based on previous findings and expected medium to large effect sizes for the within-subjects design [8, 37].

## Results

### Perception of missing beats

The effect of Rhythm (Simple, Complex) on missing tone identification in the Perception condition was tested with paired t-tests. There were no significant differences between Rhythm conditions for the hit rate (*t*(24) = .681, *p* = .251, *Cohen’s d* = .138). Participants were equally good at detecting missing tones in the Simple rhythm (90.4%) and the Complex rhythm (88.0%). Similarly, there were no significant differences between rhythm conditions for the false alarm rate (*t*(24) = 0.0, *p* = .50, *Cohen’s d* = 0.0). Participants did not make more false detections in the Complex rhythm (7.2%) than the Simple rhythm (7.2%). Thus, the analyses supported a null hypothesis of no perceptual differences between the Simple and Complex rhythms.

### Synchronization of taps

To confirm that participants maintained the same overall tapping rate across conditions, we first examined the mean intertap intervals (ITI) across Rhythms (Simple, Complex) in the Synchronization condition. As expected for the fixed tapping rates across stimuli in the Rhythm conditions, participants’ mean ITIs did not differ significantly between the Simple and Complex rhythms (*F*(1,24) = .522, *p* = .477, *generalized η*^*2*^ = .009). Thus, the results were consistent with the null hypothesis that there were no differences in tapping rate across Simple and Complex rhythms (and therefore, any asynchrony differences by Rhythm condition are not a function of differences in tapping rates).

**Fig 2:**
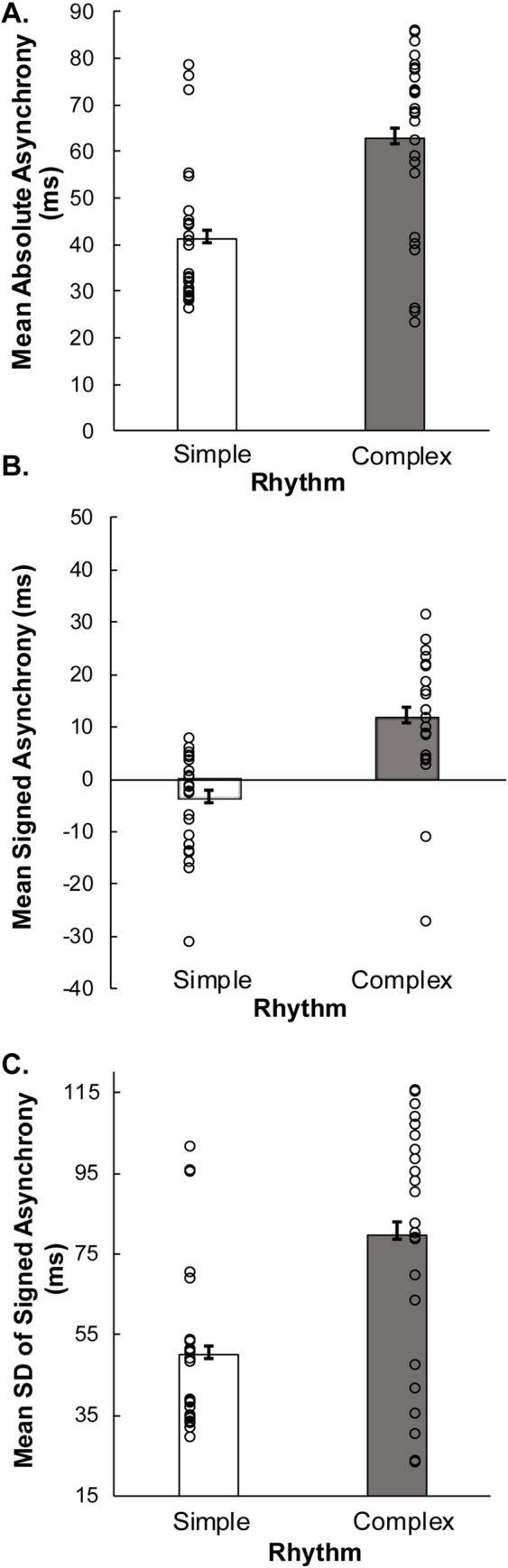
Behavioral synchrony measures by rhythm condition in the synchronization task. A): Mean absolute asynchrony by Rhythm condition (in ms). B): Mean signed asynchrony by Rhythm condition (in ms). C): Mean standard deviation of signed asynchrony by Rhythm condition (in ms). Circles = individual participants’ values.

Next, we assessed participants’ tapping synchronization accuracy in the Synchronization conditions. As shown in Fig 2A, mean absolute asynchrony values were significantly larger for the Complex rhythm than the Simple rhythm (*F* (1,24) = 21.1, *p* < .001, *generalized η*^*2*^ = .278. As shown in Fig 2B, the Complex rhythm yielded significantly larger positive signed asynchrony values whereas the Simple rhythm yielded smaller negative values (*F* (1,24) = 25.46, *p* < .001, *generalized η*^*2*^ = .330). These results constitute support for the alternative hypothesis that synchronization accuracy is worse for Complex rhythms than for Simple Rhythms.

Finally, analyses of the synchronization tapping variability were conducted in the Synchronization condition. The standard deviation of the signed asynchronies was significantly larger for the Complex rhythm than the Simple rhythm (*F* (1,24) = 18.5, *p* < .001, *generalized η*^*2*^ = .255), as shown in Fig 2C, supporting the alternative hypothesis that synchronization precision was worse for the Complex rhythm than for the Simple rhythm. Overall, these results indicate that participants synchronized their taps less accurately and less precisely with the Complex rhythm, and they showed greater anticipation (ie, their taps preceded the stimulus more) with the Simple rhythm.

### Linear measures of cardiac rhythms

We first compared the linear cardiac activity measures between Baseline (at rest) with the cardiac activity during the auditory rhythm tasks, to confirm that the presence of auditory rhythms was responsible for cardiac changes. A one-way ANOVA on mean R-R intervals by Task (Baseline, Perception, Synchronization) revealed a significant effect of Task (*F*(2,48) = 5.280, *p* = .008, *generalized η*^*2*^ = .012). The R-R intervals were larger (heart rate was slower) during the Perception condition (mean = 768.335 ms) compared to Baseline (mean = 744.161 ms) (Holm-Bonferroni corrected *t*(24) = 3.389, *p* = .002, *Cohen’s d =* .26) and during Perception compared to Synchronization (mean = 745.052 ms) (*t*(24) = 3.332, *p* = .003, *Cohen’s d =* .24). There was no significant difference between the Baseline and Synchronization conditions (*t*(1,24) = -.084, *p* = .934, *Cohen’s d =* .01). A one-way ANOVA on RMSSD by Task (Baseline, Perception, Synchronization) yielded no significant differences (*F*(2,48) = 1.360, *p* = .267, *generalized η*^*2*^ = .005), supporting the null hypothesis of no task effect on heart rate variability. Thus, the R-R intervals but not the HRV differed between Baseline (no auditory rhythms) and other tasks (auditory rhythms present).

To test for task and rhythm complexity effects, we conducted two-way ANOVAs on the linear cardiac measures (R-R inteval and RMSSD) by Task (Perception, Synchronization) and Rhythm (Simple, Complex). The ANOVA on mean R-R intervals showed a significant main effect of Task (*F* (1,24) = 11.103, *p* = .003, *generalized η*^*2*^ = .014), confirming that heart rates were slower during Perception than during Synchronization for both Simple and Complex rhythms. There were no significant effects of Rhythm or Task x Rhythm interaction. The two-way ANOVA on RMSSD indicated a significant main effect of Task (*F*(1,24) = 5.609, *p* = .026, *generalized η*^*2*^ = .075). There was greater heart rate variability during Perception than during Synchronization for both Simple (Perception = 34.78 ms, Synchronization = 32.17 ms) and Complex rhythms (Perception = 34.49, Synchronization = 31.37). There were no other significant main effects or interactions. Overall, the linear cardiac findings indicate that heart rate was faster and less variable during the Synchronization condition compared to the Perception condition, and no significant effects of rhythm complexity.

### Nonlinear measures of cardiac rhythms

We first compared the nonlinear cardiac metrics for the Baseline condition (at rest) with the cardiac activity during the auditory rhythm tasks, to confirm that the cardiac measures differed in the presence of auditory rhythms (tasks) from the absence of auditory rhythms (Baseline). For Recurrence Rate, there was a significant effect of Task (*F*(1,24) = 10.40, *p* < .001, *generalized η*^*2*^ = .119), with greater cardiac recurrence during Perception (mean = 3.062%) than Baseline (mean = 1.803%) (pairwise comparisons, Holm-Bonferroni corrected *t*(24) = 4.156, *p* < .001, *Cohen’s d =* .96) and during Synchronization (mean = 3.283%) than Baseline (*t*(24) = 4.286, *p* < .001, *Cohen’s d =* .93). Perception and Synchronization conditions did not differ significantly (t(24) = -.546, *p* = .590, *Cohen’s d =* .08). These analyses demonstrate support for the alternative hypothesis that Task affects Recurrence Rate. Determinism values, based on fixed recurrence rates (5%), showed similar main effects of Task (*F*(1,24) = 4.395, *p* = .018, *generalized η*^*2*^ = .027), indicating support for the alternative hypothesis that Task affects cardiac Determinism. Follow-up pairwise comparisons (Holm-Bonferroni corrected) indicated greater Determinism during Perception (mean = 65.71%) than Baseline (mean = 62.51%) (*t*(24) = 2.862, *p* = .009, *Cohen’s d =* .37) and during Perception than Synchronization (mean = 62.85%) (*t*(24) = 2.486, *p* = .02, *Cohen’s d =* .36). There was no significant difference between the baseline and Synchronization conditions (*t*(24) = -.267, *p* = .792, *Cohen’s d =* .04). Thus, the nonlinear cardiac measures differed between the Baseline condition (no auditory rhythms) and the other tasks (auditory rhythms present).

**Fig 3:**
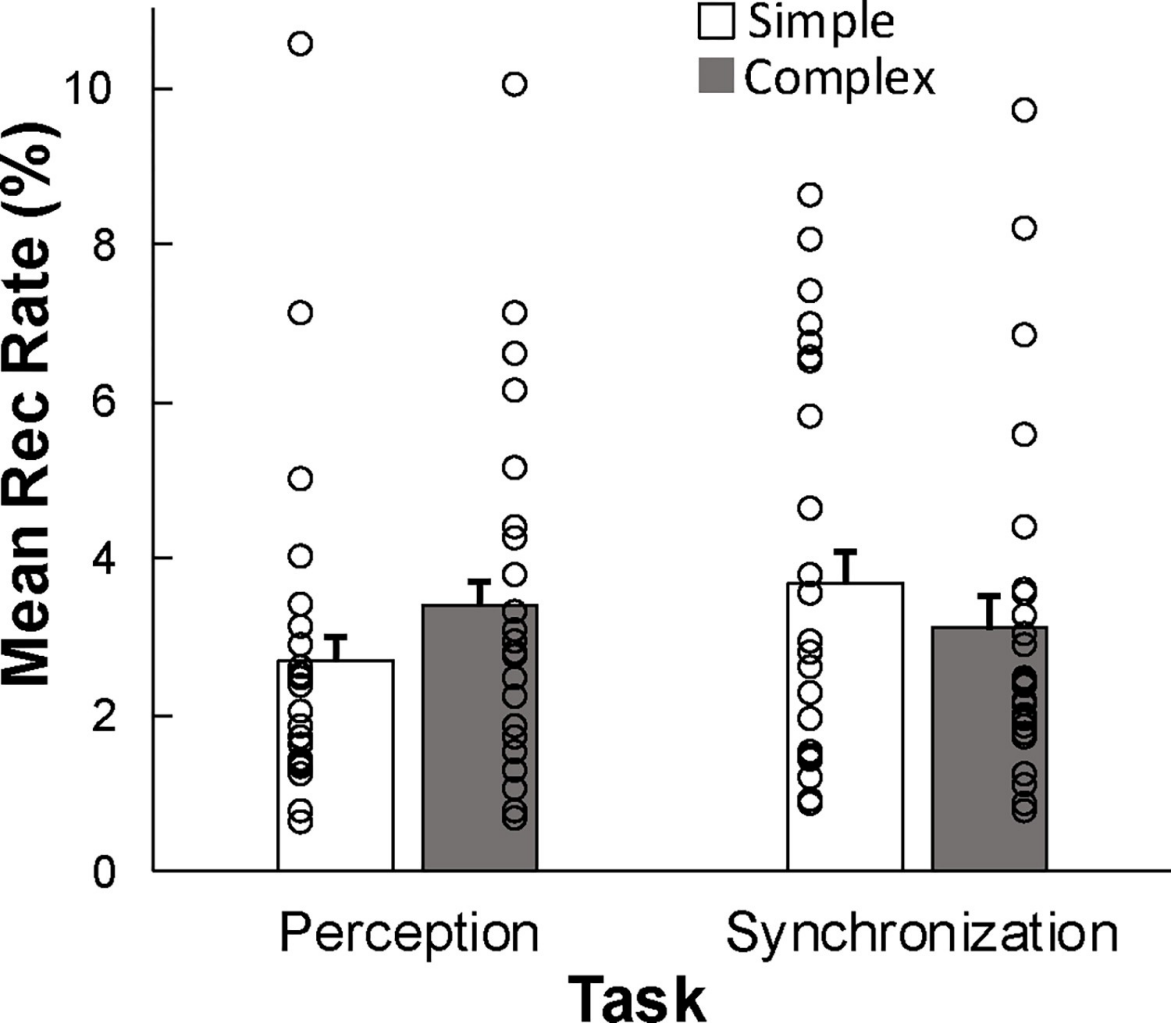
Mean recurrence rate (%) in the cardiac time series (beats per minute) by task (perception, synchronization) and rhythm (simple, complex). Circles = individual data points.

**Fig 4:**
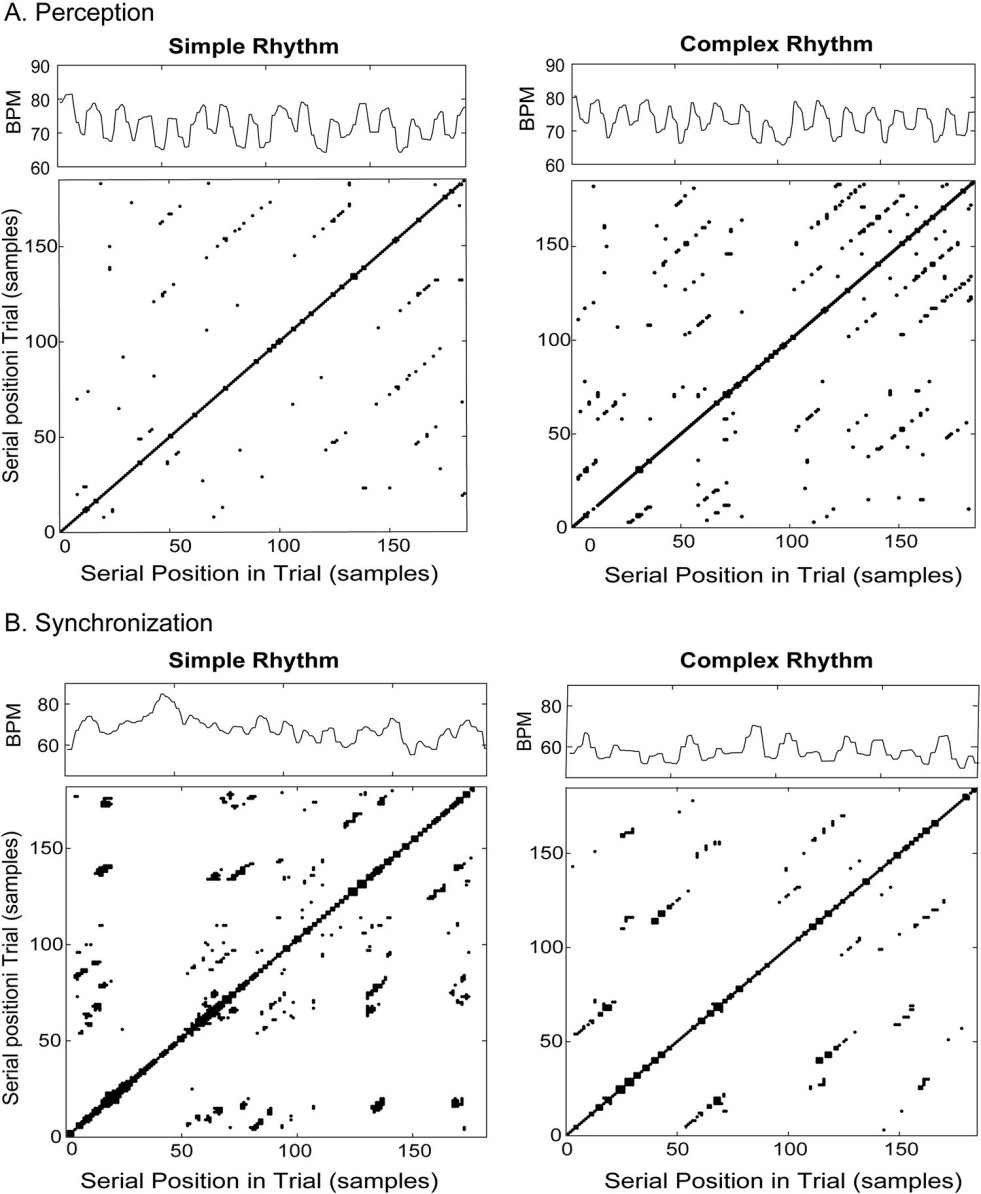
Sample cardiac time series (in beats per minute) and recurrence plots (in number of samples) from one participant for each auditory condition and rhythm condition. A) Perception task: Simple rhythm (left) and Complex rhythm (right). B) Synchronization task: Simple rhythm (left) and Complex rhythm (right). Black dots in recurrence plots indicate points of recurring heartbeat intervals.

To test for auditory rhythm effects on the nonlinear cardiac measures, a 2-way ANOVA on Recurrence Rate was conducted by Task (Perception, Synchronization) and Rhythm (Simple, Complex). This yielded a significant Task x Rhythm interaction (*F*(1,24) = 11.542, *p* = .002, *generalized η*^*2*^ = .022), and no main effects. There was significantly more recurrence during perception of the Complex rhythm than during perception of the Simple rhythm, and significantly more recurrence during synchronization with the Simple rhythm compared to synchronization with the Complex rhythm (*HSD* = .039, *p* < .05; Fig 3). Fig 4 shows example recurrence plots for the same individual’s trials of Simple and Complex rhythms from the Perception and Synchronization tasks. The recurrence plot for Complex Rhythm Perception contains more recurrent points (black dots, greater Recurrence Rate) than does the plot for Simple Rhythm Perception by the same individual. The Synchronization condition plots show the opposite pattern: Synchronization with the Simple rhythm leads to more recurrent points than does Synchronization with the Complex rhythm.

**Fig 5:**
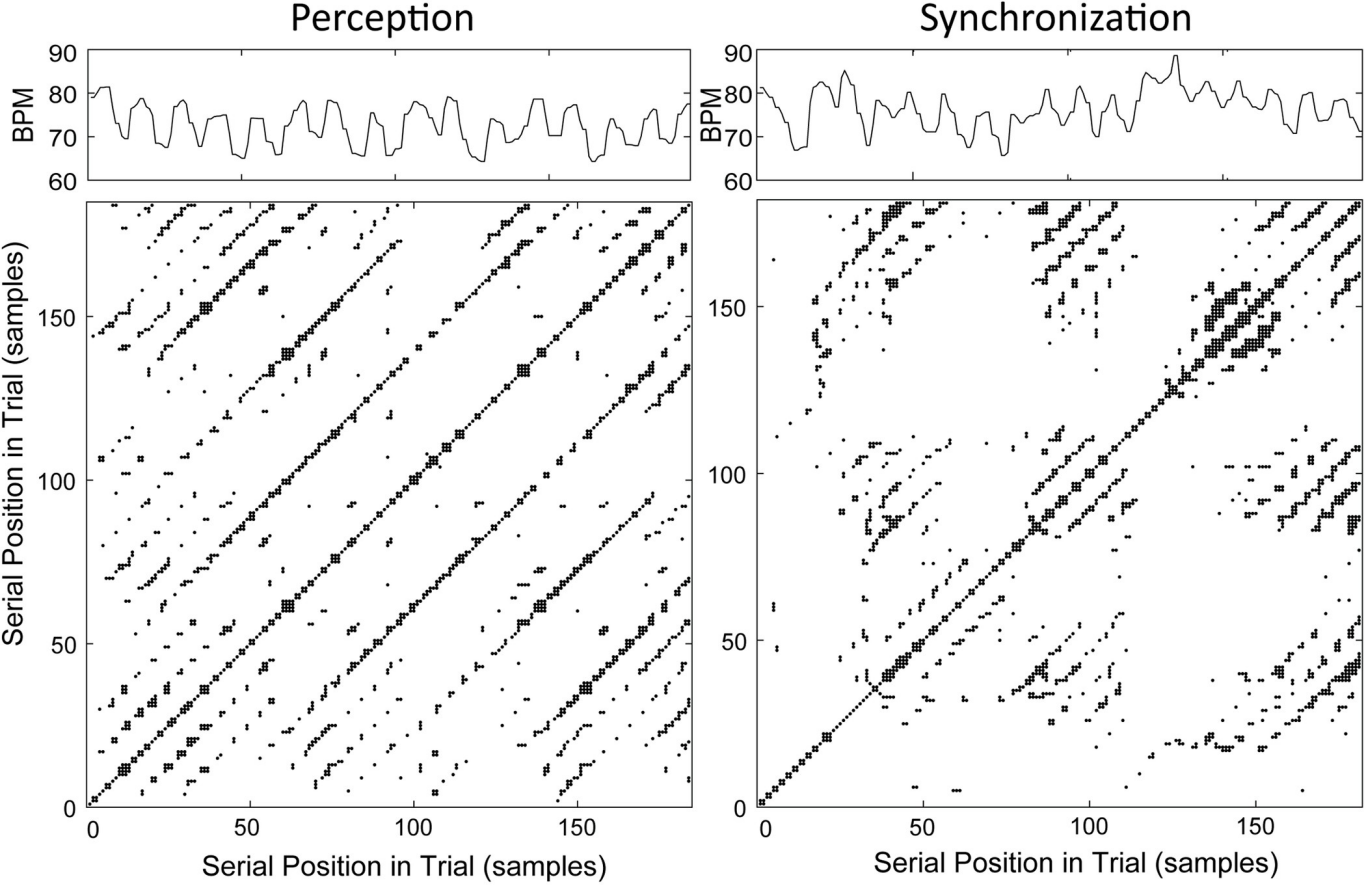
A) Mean % Determinism in the cardiac time series (beats per minute) by Task. Circles = individual data points. B) Sample cardiac time series (in beats per minute) and recurrence plots (in number of samples) from one participant for each Auditory Condition with the Simple rhythm. Left) Perception task. Right) Synchronization task. Diagonal lines of black dots indicate determinism (predictability), sequential recurring heartbeat intervals in the cardiac time series.

The same two-way ANOVA on Determinism showed a significant main effect of Task (*F*(1,24) = 6.107, *p* = .021, *generalized η*^*2*^ = .029) with greater cardiac determinism (more predictability) during rhythm Perception than during Synchronization for both Simple (Perception = 65.4%, Synchronization = 63.3%) and Complex (Perception = 66.0%, Synchronization = 62.4%) auditory rhythms. This finding supports the alternative hypothesis that Task affects cardiac Determinism. There was no significant effect of Rhythm or Task x Rhythm interaction. Fig 5 shows example recurrence plots for a single participant during individual trials of the Simple rhythm in Perception and Synchronization conditions. There is greater determinism, or proportion of black dots forming diagonal lines (indicating sequential runs of recurrence in the time series), in the cardiac patterns during Perception compared to Synchronization.

### Behavior-cardiac correlations

**Fig 6:**
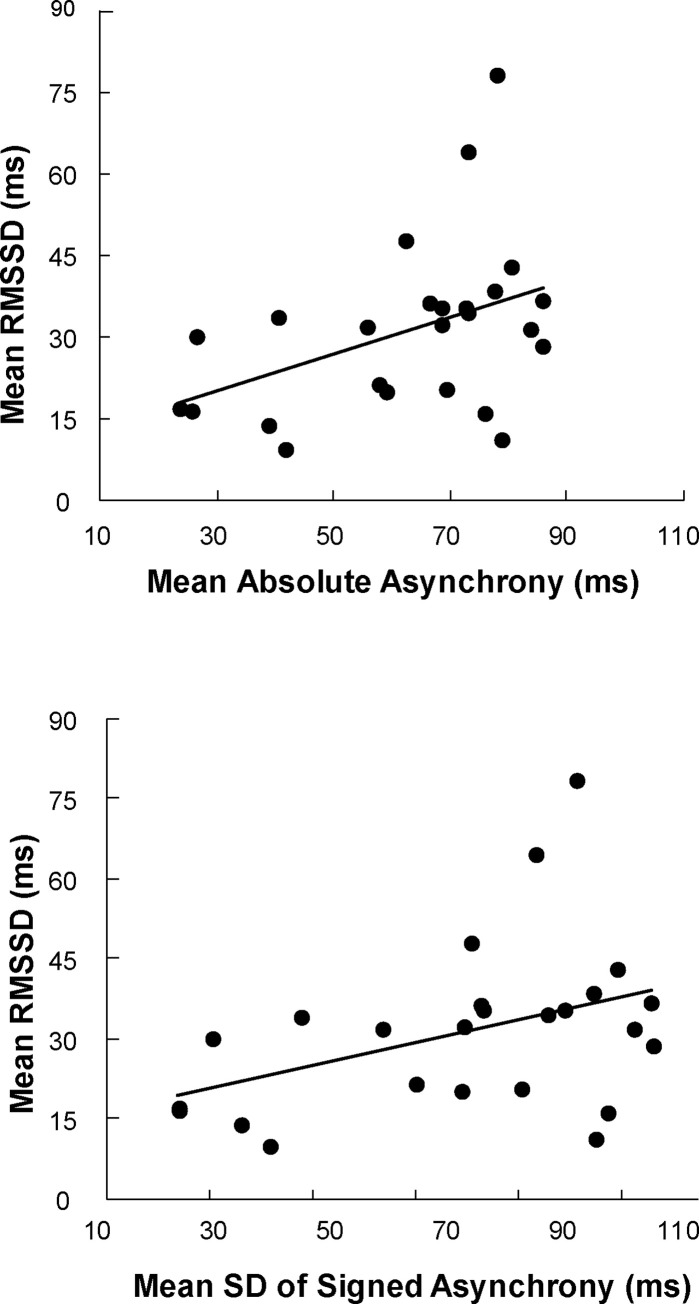
Simple correlations. A) Mean Absolute Asynchrony and RMSSD, in ms, and B) Mean Standard Deviation of Signed Asynchrony and RMSSD, in ms.

Simple correlations were used to test for individual differences in behavioral synchrony-cardiac relationships in the Synchronization condition at the participant level. We compared each participant’s mean heart rate variability (RMSSD) with mean absolute asynchrony and the standard deviations of signed asynchronies within each rhythm condition. Fig 6 shows the significant correlations observed for Complex rhythms. Participants with larger mean absolute asynchrony values (less accurate synchronization) exhibited greater RMSSD values (*r* = .41, *p* = .042). Also in the Complex rhythm condition, participants with larger standard deviations of signed asynchronies (more variable synchronization) showed larger RMSSD values (*r* = .40, *p* = .0499). The same correlations for the Simple rhythm were small and did not reach significance (mean absolute asynchrony: *r* = .05, *p* = .821; standard deviation of signed asynchronies: *r* = .001, *p* = .998). In sum, these synchronization-cardiac correspondences indicate that worsened synchronization accuracy and variability were correlated with greater heart rate variability during the Complex rhythms.

## Discussion

This study demonstrated effects of rhythm complexity on cardiac dynamics during perception and production (synchronization) of auditory rhythms. Musically trained adults (≥ 6 years private musical instruction) listened to and tapped with a Simple rhythm (that formed a 1:2 duration ratio between the two rhythms) and with a Complex rhythm (that formed a 3:2 duration ratio between the two rhythms) while their cardiac activity was measured. Linear and nonlinear analysis methods were applied to the participants’ cardiac dynamics measured during rhythm Perception and Synchronization. Listeners identified omitted tones accurately in both Simple and Complex rhythms, indicating successful perception. Complex auditory rhythms were more difficult to synchronize with than were Simple rhythms. Individual differences analyses indicated that more inaccurate and more variable synchronization corresponded to greater heart rate variability. Finally, nonlinear analyses of recurring patterns in cardiac dynamics were differentially modulated by both the task (Perception or Synchronization) as well as by stimulus rhythm complexity. Thus, nonlinear cardiac measures of recurrence rate and determinism were more sensitive to rhythm complexity than were linear measures.

Participants’ behavioral difficulties in synchronizing with Complex rhythms match predictions from dynamical systems theory [24–26], and other behavioral findings [2, 4, 5, 30, 37] indicate that synchronizing with Complex rhythm ratios (such as the 3:2 rhythms used here) is more difficult than with Simple ratios (such as the 1:2 rhythms used here). Tappers in the current study showed less accurate and less precise synchronization of taps with the Complex rhythm than the Simple rhythm. Importantly, tapping rates were held constant between the Simple and Complex rhythms, and thus motor system demands were equated and cannot account alone for the poorer performance on the Complex rhythm. There was also less anticipatory synchrony for the Complex rhythm than the Simple rhythm, reflected in taps that lagged the stimulus tones for the Complex rhythm. Notably, there were no rhythm complexity effects for the missing tone detection task in the Perception condition, suggesting that the observed synchronization differences were not simply due to differences in perceptual processing of the Simple and Complex rhythms. It may be that rhythm complexity affects production more than perception because auditory-motor coupling is required, yielding greater task difficulty.

Perception/production differences in cardiac rhythms indicated that cardiac activity was modulated by auditory perception and synchronization tasks, above and beyond the complexity of the rhythm. Rhythmic synchronization resulted in faster heart rates (smaller R-R intervals) and decreased heart rate variability compared to rhythm perception. These findings are consistent with previous findings of faster heart rates and decreased heart rate variability when pianists performed a complex musical piece compared to when they listened to the same piece [62]. The Synchronization task may have been more difficult and resulted in greater physiological arousal, as it required individuals to simultaneously perceive one rhythm while producing another auditory rhythm. More difficult cognitive and motor tasks have been reported to result in increased autonomic arousal [9], including in the auditory domain [11]. Similar physiological patterns have also been observed with increased physical effort during exercise [63], leading to reductions in heart rate variability and complexity in the cardiac signal [64]. A related interpretation is that greater physiological arousal in the Synchronization condition reflected performance anxiety, leading to the observed changes in heart rate and heart rate variability [23, 41]. However, the linear cardiac metrics observed here were similar between the Baseline (no auditory stimulus) and Synchronization conditions suggesting that performance anxiety cannot account for the observed differences.

Nonlinear measures of cardiac rhythms, including recurrence rates and determinism (predictability), showed that cardiac dynamics were more predictable during rhythm perception than during synchronization. These findings contrast with previous reports of increased cardiac predictability accompanying increased task difficulty [46, 50, 65]. Notably, Wright and Palmer [8] reported increased cardiac predictability when musicians performed unfamiliar (novel) musical rhythms compared to familiar musical rhythms; that study did not compare perception and performance conditions. The design of the current study allowed us to dissociate cardiac effects of simply perceiving auditory rhythms from perceiving while simultaneously producing the same rhythms. In the Perception task, the timing of the stimulus rhythms was computer-generated with no variability; in the Synchronization task, the timing of the auditory rhythms contained variability as participants produced one part of the rhythm. These findings thus suggest that nonlinear properties of cardiac dynamics may be modulated by stimulus timing and rhythmic motor timing.

Effects of rhythmic timing on cardiac dynamics were also evidenced in the striking Task (perception/synchronization) by Rhythm (simple/complex) interaction observed for cardiac recurrence rate (indicating repeating patterns). There was greater cardiac recurrence during Perception of the Complex rhythm (which presented different temporal intervals between the two parts), and greater cardiac recurrence during Synchronization for the Simple rhythm. The order of the conditions (Perception, then Synchronization) and rhythms (Simple, then Complex) was constant across participants, suggesting that the Task by Rhythm interaction cannot be explained simply by order effects. It also cannot be explained by participants’ tapping rates between conditions, as no tapping rate differences were observed. The RQA findings also do not fit with a straightforward interpretation of increased physical stress, which suggests decreases in physiological complexity as observed by lower heart rate variability and sample entropy [66] and greater cardiac recurrence and determinism [67]. It is possible that cardiac activity was less recurrent during Synchronization with the Complex rhythm due to increased motor noise associated with participants’ more variable asynchronies in this condition. Finally, cardiac recurrence may not reflect the same underlying causes in perception and synchronization/production of auditory rhythms, as motor timing and stimulus timing do not individually explain the observed interaction. Future research may disentangle this explanation by investigating how tapping rhythms in the absence of auditory feedback affects cardiac dynamics.

Correlations between individuals’ synchronization performance and linear cardiac measures support the interpretation that cardiac dynamics are sensitive to auditory and motor timing. Participants’ lower accuracy and more variable synchronization was correlated with increased heart rate variability in the Complex rhythm condition only. This is interesting because poorer performance indicates that synchronizing with the Complex rhythm was more difficult, yet heart rate variability increased, the opposite of task difficulty effects [68, 69]. Notably, those tasks did not manipulate the sequential stimulus timing, a factor unique to the current design. Thus, individual differences in synchronization and heart rate variability are consistent with the interpretation that cardiac activity may reflect increased noise in auditory and motor timing.

Only nonlinear cardiac analyses were sensitive to rhythm complexity, as expected for time series analyses that derive recurrences from successive items in a time series (such as the sequential ratios between successive intertap intervals that defined the Simple and Complex rhythms). The recurrence measures were able to capture the cardiac differences between the Simple and Complex auditory rhythms. Similar findings in ensemble musicians’ joint cardiac dynamics showed increased cardiac recurrence and predictability during quartet music performance [70]. The current study suggests that nonlinear analysis methods applied to R-R cardiac intervals may be well-suited for detecting changes in cardiac rhythms during temporally patterned tasks such as auditory rhythm production.

There are a few important limitations in the findings to consider. First, musical training may influence physiological measures such as cardiac dynamics in response to auditory rhythms. Nonmusicians tend to exhibit greater variability in rhythmic tapping performance than musicians [71, 72]; comparison of musically trained with untrained individuals may reveal different relationships. Second, natural music produced in richer contexts introduces many more acoustic features such as pitch, timbre and tempo; cardiac dynamics are likely to be affected by additional features of complex musical pieces. Future research may compare Simple and Complex musical rhythms in natural musical compositions to complement the constrained basic rhythm complexity findings reported here. Natural musical settings such as concerts would allow for investigation of affective influences (valence, arousal) of rhythm complexity on listeners and performers. Finally, the relationship of the R-R cardiac signals to other cardiac metrics (such as J-T intervals; [64]) may extend the findings reported here.

Overall, this study demonstrated that auditory rhythm perception and production influence cardiac activity in musically trained individuals. Complex auditory rhythms (those that formed non-integer duration ratios) were more difficult to synchronize with than Simple auditory rhythms (those that formed integer duration ratios). Synchronization with auditory rhythms resulted in faster heart rate, decreased heart rate variability, and more predictable cardiac dynamics than perception of the same rhythms. Nonlinear cardiac dynamics showed more recurrence when participants synchronized with Simple compared to Complex auditory rhythms; participants who were worse at producing Complex rhythms showed decreased heart rate variability. To our knowledge, this is the first study to demonstrate altered cardiac dynamics while individuals perceived and synchronized with auditory rhythms varying in complexity. These findings suggest that auditory-motor coupling during rhythm production may be an important modulator of cardiac dynamics that extends beyond perceptual influences.

## Supporting information

S1 File (DOCX)
