# Supplementary material for: Auditory rhythm complexity affects cardiac dynamics in perception and synchronization
Source: PLoS One. 2023 Nov 17;18(11):e0293882. doi: 10.1371/journal.pone.0293882 (PMC10656015; doi:10.1371/journal.pone.0293882)
Supplement: S1 File — (DOCX) [file pone.0293882.s001.docx]

**Supporting Information**

**S1 Appendix**

Recurrence quantification analysis (RQA) was used as the nonlinear analysis method of cardiac activity. In contrast to the linear cardiac measures, auto-RQA captures cardiac dynamics - or changes in the heart’s activity over time. A time-delayed copy of the normalized cardiac time series is produced to reconstruct the signal in a multi-dimensional phase space [1, 2]. A time delay parameter and an embedding dimension parameter are used to yield the reconstructed phase space. In the current study, the time delay was set to the first local minimum of the average mutual information function [3]. The time delay values at the trial level ranged from 4-29. The embedding dimension parameter, which determines the dimensionality of the phase space, was chosen using the False Nearest Neighbours method [3] in which the first local minimum was selected as the embedding dimension for each trial. Embedding dimensions ranged from 4-17. The time delay and embedding dimension values across trials and participants were fixed to the median of the observed values (time delay = 4, embedding dimension = 4). This prevents individual- and condition-level differences in the dependent variables from being artefacts of the parameters varying across individuals and/or conditions [3]. Figure S1 (top) shows an example of a phase space trajectory of a cardiac signal with a time delay = 4 and embedding dimension = 3.

Points in the phase space are then assessed for their closeness using a radius threshold parameter; points that are sufficiently close to one another in the phase space are deemed to be recurrent [3]. Recurrent points are then plotted in a 2-dimensional, binary (black=recurrent, white=not recurrent) auto-recurrence plot (Figure S1 bottom). We set the radius to 10% of the diameter of the phase space for the recurrence rate analysis [3], which allowed recurrence plots to vary in the proportion of recurrent points. For analyses on cardiac determinism, we held the recurrence rate constant at 5% to distinguish determinism effects beyond simple recurrence [4, 5].

Finally, points in the recurrence plot can be quantified according to different metrics to describe the behavior of the cardiac system. Those metrics in the current study included Recurrence Rate and Determinism. Recurrence rate is the proportion of recurrent points in a time series and represents how often the system returns to a previous state [2]. Determinism measures the proportion of recurrent points that occur sequentially in the time series (forming diagonal lines in the recurrence plot) and represents the predictability of the system [3, 6, 7].


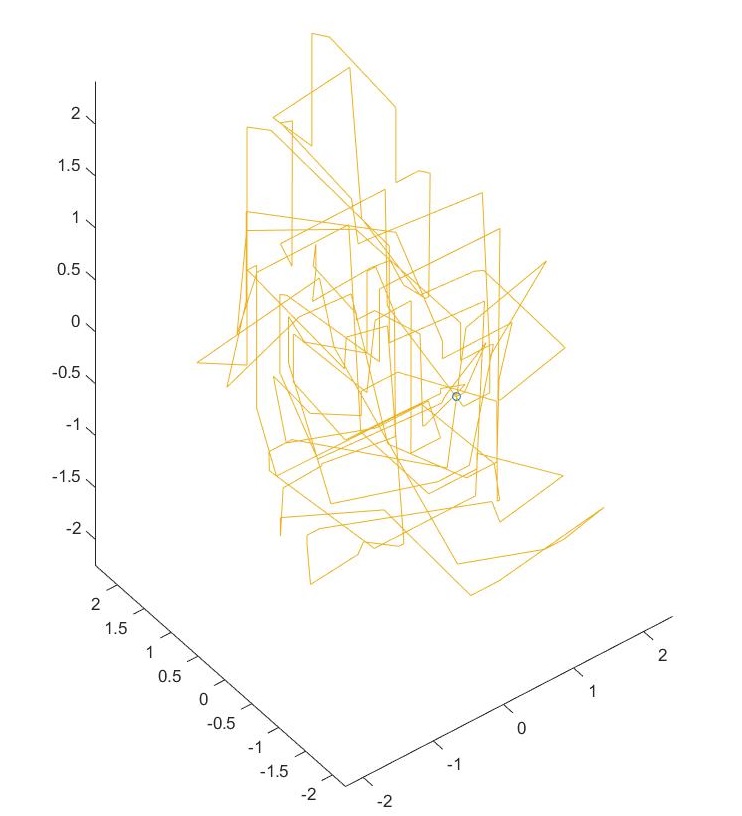


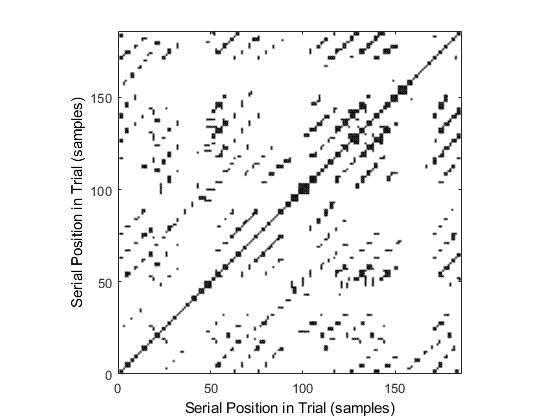


**Fig S1.** **Example phase space trajectory and recurrence plot.** Top: Example of a single participant’s phase space trajectory of cardiac activity for a 1-minute tapping trial with embedding dimension = 3 and time delay = 4. Bottom: The recurrence plot derived from the phase space. The cardiac time series is plotted on the x- and y-axes. Each black dot represents a point that was recurrent with another point in the phase space. White space represents non-recurrent points. The total proportion of black points in the recurrence plot is the Recurrence Rate. Diagonal structures represent sequential points of recurrence, signalling cardiac predictability. The proportion of black points that form diagonal lines is Determinism (%).

**References**

1. Takens, F. Detecting strange attractors in turbulence. In Rand D, Young L. (Eds.), Dynamical Systems and Turbulence. Berlin: Springer; 1981. pp. 366-381
2. Webber Jr CL, Zbilut JP. Recurrence quantification analysis of nonlinear dynamical systems. in: Riley MA, Van orden GC. Tutorials in contemporary nonlinear methods for the behavioral sciences. National Science Foundation; 2005. pp. 26-94.
3. Webber CL, Marwan N. Recurrence quantification analysis. Theory and Best Practices. Spring International Publishing: Switzerland. 2015.
4. Javorka M, Turianikova Z, Tonhajzerova I, Javorka K, Baumert M. The effect of orthostasis on recurrence quantification analysis of heart rate and blood pressure dynamics. Physiol Meas. 2009; 30(1): 29-41.
5. Javorka M, Trunkvalterova Z, Tonhajzerova I, Lazarova Z, Javorkova J, Javorka K. Recurrences in heart rate dynamics are changed in patients with diabetes mellitus. Clin Physiol Funct Imaging. 2008; 28(5): 326-31.
6. Marwan N, Carmen Romano M, Thiel M, Kurths J. Recurrence plots for the analysis of complex systems. Phys Rep. 2007; 438(5-6): 237-329.
7. Nayak SK, Bit A, Dey A, Mohapatra B, Pal K. A review on the nonlinear dynamical system analysis of electrocardiogram signal. J Healthc Eng. 2018 May 2. doi: 10.1155/2018/6920420.
